# The Actress, the Court, and What Needs to Be Done to Guarantee the Future of Clinical Genomics

**DOI:** 10.1371/journal.pbio.1001663

**Published:** 2013-09-24

**Authors:** Arthur L. Caplan

**Affiliations:** Division of Medical Ethics, NYU Langone Medical Center, New York, New York, United States of America

## Abstract

Clinical genomics is poised for a rapid expansion but more work must be done to build a supporting ethical infrastructure.

On May 14 of this year, the actress Angelina Jolie wrote an essay in the *New York Times* that received enormous attention around the world. She revealed that, after receiving genetic testing and counseling, she had undergone a double mastectomy. She had the surgery even though she did not have breast cancer [1].

Ironically, shortly after her decision to go public about her prophylactic mastectomy, the United States Supreme Court issued an important ruling about the patentability of the genetic test Jolie had used [2]. Many predict that these two events will greatly increase interest in genetic testing for breast cancer and other diseases [3],[4]. That may be, but these events should also draw attention to a number of key ethical issues that remain unresolved regarding genetic testing.

If clinical genomics is about to move forward at a more rapid pace due to broader public awareness and a more favorable legal climate then there is still work to be done on the ethical, regulatory, and legal fronts [5]. While Jolie had access to testing, much still needs to be done to remedy disparities in access to testing and follow-up treatments for others at high risk due to heredity. At the same time, outside of those in known risk groups, like Jolie, it is not clear when genetic testing for breast cancer and other conditions in the general population makes sense [6]. The control and third-party use of genetic information obtained through testing remains uncertain [7]. What information ought to be disclosed to those undergoing genetic testing, by whom, and with what, if any, sort of counseling is also still unsettled [8]. What will be said when further advances in the precision of genetic testing reveal that some may have had mastectomies or removed their prostate, stomach, or ovaries unnecessarily [9]? And there still remain penalties (higher costs for life or disability insurance) facing those either identified as being at genetic risk or who simply have sought testing that need to be addressed [7].

Perhaps the most obvious question that now arises is when is clinical genetic testing appropriate for everyone? Consider testing for breast cancer. Breast cancer is the most common malignancy in women. Approximately 200,000 women receive a diagnosis of breast cancer each year in the United States. About 40,000 women die from the disease. The five-year survival rate for women with breast cancer has been improving and is currently estimated to be greater than 85% [10].

Approximately 5% to 10% of breast cancer cases are associated with a hereditary predisposition. Most of these cases are related to mutations in the *BRCA1* and *BRCA2* genes. Hereditary breast cancer syndrome is inherited in an autosomal-dominant fashion. Mutations in either *BRCA1* or *BRCA2* have been identified as the cause of these syndromes. Women who inherit these mutations have a significantly increased risk of developing breast and/or ovarian cancer.

According to the National Cancer Institute, in the general population, the incidence of breast cancer is about 0.05% per annum at age 35 and 0.2% per annum at age 50, with a cumulative lifetime risk of breast cancer of approximately 13% [10]. For women who inherit a predisposition to developing breast cancer the incidence is 1% at age 35 and 3% per annum at age 50, with a cumulative lifetime risk of approximately 80% [11].

Some, often those who sell testing, argue that every woman ought to be tested for *BRCA1* and *BRCA2* mutations [6]. The U.S. Preventive Services Task Force (USPSTF), an independent panel of experts in evidence-based medicine, recommends *BRCA* mutation testing only for women who have a family history suggestive of increased risk [11]. The yet-unresolved ethical issue is whether testing all women for a very small risk of genetically linked early-onset breast cancer is worth the resources involved, especially when there are many other important risk factors for breast cancer present for most women that are hugely determinative of cancer, not tied to genetics, and which merit attention from health care providers.

Cost has sharply limited access to testing. Jolie said she had chosen to have her breasts removed because she knew her mother and other relatives had been ravaged by breast cancer at relatively young ages. Knowing this, she had sought out genetic testing that could reveal if she was at increased risk of a hereditary form of breast cancer. She had found out, through the use of Myriad Genetics' then patented genetic test for breast cancer, that she had inherited the mutated gene and thus was at high risk for both breast and ovarian cancer. The cost of testing, which is often not covered by insurance, is not insignificant—ranging from $3,000 to $4,000. The reason for the high cost is that Myriad, the Utah-based provider of the test, has long had a monopoly over its sale [6],[12],[13].

Almost four years to the day before Jolie's revelation, Myriad had been the target of a highly unusual lawsuit. A variety of clinics and organizations sued, challenging the validity of patents Myriad had received for identifying the gene mutations associated with a greater risk of breast cancer. On May 12, 2009, a group of plaintiffs led by the American Civil Liberties Union and the Public Patent Foundation filed a lawsuit, *Association for Molecular Pathology, et al. v. U.S. Patent and Trademark Office, et al.*, against Myriad Genetics and the U.S. Patent and Trademark Office [13]. The lawsuit contended that the patents granted on two human gene variations associated with breast and ovarian cancer were invalid, unconstitutional, and hindered access to testing. If a woman wanted to know whether her genes contained cancer-causing mutations, the only way to find out was through a test that required paying Myriad's monopolistic, patent-protected fee.

On April 15, 2013, just a few weeks before the Jolie revelation, the Supreme Court heard oral arguments about the patent case. On June 13, the Court ruled that isolated genomic DNA is not patent-eligible under section 101 of the Patent Act [2]. The Court struck down patent claims over any DNA that had merely been “isolated” from cells—removed from its natural environment in the cell nucleus and the contents analyzed—exactly what Myriad had done using the prior work of University of Washington geneticist Mary-Claire King and her team. Her team had first discovered the association between key mutations at *BRCA* genetic loci and a higher risk of breast cancer [14],[15].

From an ethical point of view, striking down the claims in Myriad's patents was the right thing to do. Aside from isolating and removing genetic material, the patent merely laid claim to natural genetic sequences. This did not constitute a useful invention in that nothing was constructed, invented, or manipulated [12] Myriad's claims amounted to looking through the first telescope at the planets and their moons and seeking to patent them. Ethically and legally, what exists in nature should not be subject to any patent claim.

The court rightly made way for patent claims that involve manipulating genes, altering them, synthesizing new ones, or simplifying existing genes into more useful constructs. In doing so, the court secured the future of the biotechnology industry that is rapidly expanding along all of these lines of genetic research in humans, animals, plants, and microbes.

The link between Jolie's decision and the patent fight settled one pressing question—can you patent what exists in nature? The decision has been hailed as opening the door to cheaper genetic testing for breast cancer and for other genetic risk factors for disease [3],[4],[16]. If the termination of Myriad's patents does indeed open the door to more and cheaper forms of clinical genetic testing, it also opens the door to a number of other important moral questions that must be resolved if testing is to flourish.

The rapid expansion of genetic testing beyond breast cancer in high-risk adults to other women would potentially not be cost-efficient given the relative rarity of the gene mutations involved in the general population but would also strain the existing resources for genetic counseling. If testing expands to include testing for many more conditions, as well as testing fetuses by blood samples drawn from pregnant women, preimplantation genetic diagnosis of embryos, newborns [17], and more extensive carrier testing, that would surely overwhelm the currently available, properly trained supply of persons able to provide counseling.

More widespread testing also means the discovery of more incidental but important findings. There are no agreed-upon standards about how to handle incidental clinical discoveries, which can include questions about paternity and the suspicion of incest [8],[18].

Will testing information be coded for anonymity but in a way that still permits linkage to the identity of those tested? No agreed-upon rules exist about the morality of recontacting those who have been tested as future discoveries reveal more accurate information or new information about mitigating risk through lifestyle changes, changes in reproductive plans, or new medical therapies.

If clinical genomics can now be expected to move forward more rapidly with greater legal clarity, more affordability, and more public awareness then we need:

1. More public discussion of what level of risk would justify public reimbursement of genetic tests for whom and with what assurance of coverage for follow-up care and lifestyle coaching.
2. Agreement among providers to set a standard of care about the precision with which informed consent will be sought as part of clinical genomic testing that includes the reliability of anonymization protections, recontacting policy, handling incidental discoveries, contacting biological relatives with results and other third parties, and the use of information for research as well as diagnostic purposes.
3. A policy that requires clinical testing to always be accompanied by an offer of competent counseling.
4. A commitment to train health care providers to provide counseling for testing of embryos, fetuses, carriers, newborns, and adults.
5. Discussion of what traits and behaviors will be tested for, including an attempt to create a guideline of appropriate indications or even a diagnostic manual of clinical genomics.
6. An articulation of regulations governing liability for the use of testing that subsequently is shown to be limited or even erroneous by subsequent genetic research.
7. Some agreement on when direct-to-consumer advertising and recruitment of clients for diagnosis is appropriate and when it is obligatory such as for cancer patients.
8. An examination of the adequacy of existing laws such as the 2008 Genetic Information Non-Discrimination Act, which do not cover many emerging uses of clinical genomics including the acceptability of allowing information from clinical genetic testing to be used in life, health, and disability insurance as well as in employment decisions and in setting job eligibility requirements.

Since both financial and medical resources for conducting genetic testing are limited, does it make sense to concentrate on populations believed to be at greater risk of genetic diseases or should testing move rapidly toward routine testing for all? If the latter, then much more research will be needed to both broaden existing databases to include more diversity than currently exists [19] and to begin to understand how penetrance and context more precisely determine risk, age of onset, and severity of illness in various groups [19],[20].

Celebrities are now drawing public attention to the utility of genetic testing. With the Supreme Court decision opening the door to more and perhaps cheaper entry into the testing market, the requisite infrastructure for managing risk and the rules for handling risk information must be strengthened. Making testing more widely available will only be morally acceptable if there are rules of the road in place.

## References

[1] Jolie A (2013 May 14) My medical choice. The New York Times. Available: http://www.nytimes.com/2013/05/14/opinion/my-medical-choice.html?_r=0.

[2] Association for Molecular Pathology v. Myriad Genetics, 569 US ___ (2013)

[3] Blumenstyk G (2013 June 13) Academic scientists hail Supreme Court's rejection of gene patents. The Chronicle of Higher Education. Available: http://chronicle.com/article/Academic-Scientists-Hail/139813/.

[4] Pollack A (2013 June 14) After DNA patent ruling, availability of genetic tests could broaden. The New York Times; A16.

[5] Caplan AL (2012) Without an adequate ethical infrastructure, the road to personalized medicine will be rocky at best. Clin Pharmacol Ther 92: 411–412. 22992666 10.1038/clpt.2012.142

[6] Matloff E, Caplan AL (2008) Direct to confusion: lessons learned from marketing BRCA testing. Am J Bioeth 8: 5–9. 18726769 10.1080/15265160802248179

[7] Trottman M (2013) Should bosses have access to worker's genetic test results? The Wall Street Journal Available: http://online.wsj.com/article/SB10001424127887323664204578607611858697242.html.

[8] Wolf S, Annas GA, Elias S (2013) Patient autonomy and incidental findings in clinical genomics. Science 340: 1049–1050 doi:10.1126/science.1239119 23686341 10.1126/science.1239119 PMC3721305

[9] English B (2013) Family removes stomachs to cut cancer risk. The Boston Globe Available: http://www.bostonglobe.com/lifestyle/2013/07/22/family-members-with-mutant-gene-opt-for-stomach-removal-surgery/rpkp2im5zV9l0ae5vy6cEN/story.html.

[10] ECRI (2013) Hotline response: testing and genetic risk assessment for *BRCA* genetic mutations and development of breast and ovarian cancer. Available: https://www.ecri.org/Documents/Technology-Assessment/Testing_and_Genetic_Risk_Assessment_for_BRCA_Genetic_Mutations_and_Development_of_Breast_and_Ovarian_Cancer.pdf.

[11] USPSTF (2013) Genetic risk assessment and BRCA mutation testing for breast and ovarian cancer susceptibility. Available: http://www.uspreventiveservicestaskforce.org/uspstf/uspsbrgen.htm.

[12] Gostin LO (2013) Who owns human genes? JAMA E-pub ahead of print. doi:10.1001/jama.2013.177833 10.1001/jama.2013.177833 23877839

[13] Association for Molecular Pathology, et al. v. USTPO, Myriad Genetics, et al., 1:2009cv04515 (2009).

[14] Miki Y, Swensen J, Shattuck-Eidens D, Futreal PA, Harshman K, et al (1994) A strong candidate for the breast and ovarian cancer susceptibility gene BRCA1. Science 266: 66–71. 7545954 10.1126/science.7545954

[15] Hall J, Lee M, Newman B, Morrow J, Anderson L, et al (1990) Linkage of early-onset familial breast cancer to chromosome 17q21. Science 250: 1684–1689. 2270482 10.1126/science.2270482

[16] Cancer Letter (2013 July 26) Newcomers undercut price of BRCA test; myriad lawsuits claim patent infringement.

[17] Tarini BA, Goldenberg AJ (2012) Ethical issues with newborn screening in the genomics era. Annu Rev Genomics Hum Genet 13: 381–393. 22559326 10.1146/annurev-genom-090711-163741 PMC3625041

[18] Bunnik EM, de Jong A, Nijsingh N, de Wert GMWR (2013) The new genetics and informed consent. Bioethics 27: 248. 10.1111/bioe.12030 23718722

[19] Hayes DF, Allen J, Compton C, Gustavsen G, Leonard DGB, et al (2013) Tumor-biomarker diagnostics: breaking a vicious cycle. Sci Transl Med 5: 196. 10.1126/scitranslmed.3005950 23903752

[20] Derry JMJ, Mangravite LM, Suver C, Furia MD, Henderson D, et al (2012) Developing predictive molecular maps of human disease through community-based modeling. Nat Genet 44: 127–130. 22281773 10.1038/ng.1089 PMC3643818

